# Supplementary material for: Effects of Air Pollution on Lung Innate Lymphoid Cells: Review of In Vitro and In Vivo Experimental Studies
Source: Int J Environ Res Public Health. 2019 Jul 2;16(13):2347. doi: 10.3390/ijerph16132347 (PMC6650824; doi:10.3390/ijerph16132347)
Supplement: Supplementary file 1 [file ijerph-16-02347-s001.pdf]

Supplementary Material

Effects of air pollution on lung innate lymphoid cells: review of in vitro and in vivo experimental studies

Bertha Estrella, Elena N. Naumova , Magda Cepeda , Trudy Voortman , Peter D. Katsikis and Hemmo A. Drexhage

Resource S1. Search terms

Five databases were searched in cooperation with a medical information specialist to identify relevant studies: Embase.com, Medline Ovid, Web of science, the Cochrane Library, Google scholar)

Embase.com 1118

('air pollution'/de OR 'air pollutant'/exp OR 'air pollution indicator'/de OR 'environmental exposure'/de OR 'exhaust gas'/de OR acetylene/de OR benzene/de OR '1, 3 butadiene'/de OR 'carbon monoxide'/de OR dust/de OR ethane/de OR ethylbenzene/de OR ethylene/de OR 'airborne particle'/de OR 'nitrogen dioxide'/de OR 'particulate matter'/de OR toluene/de OR xylene/de OR 'polycyclic aromatic hydrocarbon'/exp OR combustion/de OR 'black carbon'/de OR 'volatile organic compound'/de OR 'tobacco use'/exp OR 'smoking and smoking related phenomena'/exp OR 'carbon nanotube'/de OR ((air NEAR/3 (clean*)) OR ((environment* OR personal) NEAR/6 (expos* OR toxican*)) OR pollut* OR microenvironment* OR exhaust* OR emission* OR acetylene* OR benzene* OR butadiene* OR (carbon NEXT/1 monoxide*) OR co OR carbonmonoxide* OR Coarse OR dust OR ethane OR ethylbenzene* OR ethylene* OR ethene* OR particle* OR (particul* NEAR/3 matter*) OR (nitro* NEXT/1 dioxide*) OR pm1 OR 'pm2 5' OR pm10 OR 'pm 1' OR 'pm 2 5' OR 'pm 10' OR soot OR toluene* OR xylene* OR ufp* OR 'black carbon' OR (polycyclic NEAR/3 (hydrocarbon* OR carbon*)) OR pah OR pahs OR combust* OR (volatile NEAR/3 compound*) OR VOCs OR voc OR tVOCs OR tvoc OR btex OR smoke OR smoking OR tobacco OR (carbon NEAR/3 nanotube*)):ab,ti) AND ('lymphoid cell'/exp OR 'cytokine'/exp OR 'cytokine response'/exp OR (((lymphoid) NEAR/3 cell*) OR lymphocyte* OR ilc OR ilcs OR (natural* NEAR/3 killer*) OR cytokine* OR interferon* OR ifn OR interleukin*):ab,ti) AND (asthma/exp OR 'respiratory tract infection'/exp OR 'respiratory tract inflammation'/exp OR (asthma* OR (airway* NEAR/6 (hyperreactiv* OR allerg* OR inflammat* OR immun*)) OR ((respiratory* OR airway*) NEAR/3 infect*)):ab,ti) AND ('innate immunity'/de OR (innate* OR non-specific* OR nonspecific* OR in-born OR inborn):ab,ti) NOT ([Conference Abstract]/lim OR [Letter]/lim OR [Note]/lim OR [Editorial]/lim)

Medline Ovid 1118

("air pollution"/ OR exp "Air Pollutants"/ OR "environmental exposure"/ OR "Vehicle Emissions"/ OR acetylene/ OR benzene/ OR "Benzene Derivatives"/ OR "butadienes"/ OR "carbon monoxide"/ OR dust/ OR ethane/ OR ethylenes/ OR "nitrogen dioxide"/ OR "particulate matter"/ OR toluene/ OR "Polycyclic Hydrocarbons, Aromatic"/ OR ((air ADJ3 (clean*)) OR ((environment* OR personal) ADJ6 (expos* OR toxican*)) OR pollut* OR microenvironment* OR exhaust* OR emission* OR acetylene* OR benzene* OR

butadiene* OR (carbon ADJ monoxide*) OR co OR carbonmonoxide* OR Coarse OR dust OR ethane OR ethylbenzene* OR ethylene* OR ethene* OR particle* OR (particul* ADJ3 matter*) OR (nitro* ADJ dioxide*) OR pm1 OR "pm2 5" OR pm10 OR "pm 1" OR "pm 2 5" OR "pm 10" OR soot OR toluene* OR xylene* OR ufp* OR "black carbon" OR (polycyclic ADJ3 (hydrocarbon* OR carbon*)) OR pah OR pahs OR combust* OR (volatile ADJ3 compound*) OR VOCs OR voc OR tVOCs OR tvoc OR btex OR smoke OR smoking OR tobacco OR (carbon ADJ3 nanotube*)).ab,ti.) AND (exp "Lymphocytes"/ OR exp "Cytokines"/ OR (((lymphoid) ADJ3 cell*) OR lymphocyte* OR ilc OR ilcs OR (natural* ADJ3 killer*) OR cytokine* OR interferon* OR ifn OR interleukin*).ab,ti.) AND (asthma/ OR "respiratory tract infection"/ OR "respiratory tract inflammation"/ OR (asthma* OR (airway* ADJ6 (hyperreactiv* OR allerg* OR inflammat* OR immun*)) OR ((respiratory* OR airway*) ADJ3 infect*)).ab,ti.) AND ("Immunity, Innate"/ OR (innate* OR non-specific* OR nonspecific* OR in-born OR inborn).ab,ti.) NOT (letter OR news OR comment OR editorial OR congresses OR abstracts).pt.

Cochrane *3*

(((air NEAR/3 (clean*)) OR ((environment* OR personal) NEAR/6 (expos* OR toxican*)) OR pollut* OR microenvironment* OR exhaust* OR emission* OR acetylene* OR benzene* OR butadiene* OR (carbon NEXT/1 monoxide*) OR co OR carbonmonoxide* OR Coarse OR dust OR ethane OR ethylbenzene* OR ethylene* OR ethene* OR particle* OR (particul* NEAR/3 matter*) OR (nitro* NEXT/1 dioxide*) OR pm1 OR 'pm2 5' OR pm10 OR 'pm 1' OR 'pm 2 5' OR 'pm 10' OR soot OR toluene* OR xylene* OR ufp* OR 'black carbon' OR (polycyclic NEAR/3 (hydrocarbon* OR carbon*)) OR pah OR pahs OR combust* OR (volatile NEAR/3 compound*) OR VOCs OR voc OR tVOCs OR tvoc OR btex OR smoke OR smoking OR tobacco OR (carbon NEAR/3 nanotube*)):ab,ti) AND ((((lymphoid) NEAR/3 cell*) OR lymphocyte* OR ilc OR ilcs OR (natural* NEAR/3 killer*) OR cytokine* OR interferon* OR ifn OR interleukin*):ab,ti) AND ((asthma* OR (airway* NEAR/6 (hyperreactiv* OR allerg* OR inflammat* OR immun*)) OR ((respiratory* OR airway*) NEAR/3 infect*)):ab,ti) AND ((innate* OR non-specific* OR nonspecific* OR in-born OR inborn):ab,ti)

Web of science *481*

TS=((((air NEAR/2 (clean*)) OR ((environment* OR personal) NEAR/5 (expos* OR toxican*)) OR pollut* OR microenvironment* OR exhaust* OR emission* OR acetylene* OR benzene* OR butadiene* OR (carbon NEAR/1 monoxide*) OR co OR carbonmonoxide* OR Coarse OR dust OR ethane OR ethylbenzene* OR ethylene* OR ethene* OR particle* OR (particul* NEAR/2 matter*) OR (nitro* NEAR/1 dioxide*) OR pm1 OR "pm2 5" OR pm10 OR "pm 1" OR "pm 2 5" OR "pm 10" OR soot OR toluene* OR xylene* OR ufp* OR "black carbon" OR (polycyclic NEAR/2 (hydrocarbon* OR carbon*)) OR pah OR pahs OR combust* OR (volatile NEAR/2 compound*) OR VOCs OR voc OR tVOCs OR tvoc OR btex OR smoke OR smoking OR tobacco OR (carbon NEAR/2 nanotube*))) AND ((((lymphoid) NEAR/2 cell*) OR lymphocyte* OR ilc OR ilcs OR (natural* NEAR/2 killer*) OR cytokine* OR interferon* OR ifn OR interleukin*)) AND ((asthma* OR (airway* NEAR/5 (hyperreactiv* OR allerg* OR inflammat* OR immun*)) OR ((respiratory* OR airway*) NEAR/2 infect*))) AND ((innate* OR non-specific* OR nonspecific* OR in-born OR inborn))) AND DT=(article)

Google scholar

Pollution|pollutant|pollutants|exhaust|"particulate matter"|pah|pahs|combustion|"black carbon"|vacs
"lymphoid cell|cells"|lymphocyte|lymphocytes|cytokine|cytokines|interferon|interferons|asthma
innate|"non-specific"|nonspecific|inborn

Resource S2. Methodological details of the studies

Author (year) Reference	Specie/ Analysis Unit	Exposure/ Dose/ route	Experimentation arm/groups	Outcomes	Findings	Conclusions
Breamer C.A, et al. 2013	Mice<br><br>Groups<br><br>*in vivo*<br>*in vitro*	Multi-walled carbon nanotubes,<br>Dose: 50 μg,<br>Route: Oropharyngeal	1. MWCNT C57Bl/6 mice<br>2. Controls (IL-13−/− and IL-33−/− mice)	• Assessment of pulmonary inflammation	MWCNT:<br>• Elevated levels of IL-33 in the lavage fluid<br>• Recruitment of ILCs in the airways.<br>• ILCs acting in response to IL-33 stimulate AHR and eosinophil recruitment through the release of IL-13.	MWCNT induce epithelial damage that results in release of IL-33, which in turn promotes innate lymphoid cell recruitment and the development of IL-13-dependent inflammatory response
				• Cytokine/chemokine (Eotaxin, IL-5, IL-6, and IL-33)	MWCNT:<br>• Induce secretion of IL-33 by epithelial cells (type II pneumocytes) twenty-four hours after exposure<br>• FA21 MWCNT and rmIL-33 result in elevated levels of eotaxin, IL-5, and IL-6.	
Mathews J, et al. 2016	Mice<br><br>Groups<br><br>in vitro	O_3<br>Dose:<br>2 ppm for 3 hrs<br>Route: Inhaled	1. Ozone db/db ((lacking leptin) obese<br>2. Ozone WT (C57BL/6J)<br>3. AIR db/db obese<br>4. TCRδ-/- vs WT	• IL-33 levels BAL	Ozone in Obese mice vs. lean WT mice<br>• Increase of BAL IL-33, neutrophils, and airway responsiveness.<br>• Increased lung IL-13+ innate lymphoid cells type 2 (ILC2) and IL-13+ γδ T cells.<br>• increased ST2+γδ T cells, indicating that these cells can be targets of IL-33,<br>• Equal decrease of serum IL-33 and was approximately 50% lower after O3 than air in both WT and obese mice	IL-33 contributes to augmented responses to ozone in obese mice. Obesity and ozone also interacted to promote type 2 cytokine production in γδ T cells and ILC2 in the lungs, which may contribute to the observed effects of IL-33.
				• IL-33 dependent BAL cytokines and chemokines	• Increase in BAL concentrations of IL-5, IL-13, and IL-9.<br>• Increases in BAL CXCL1, IL-6, IL-2, eotaxin (CCL11), CSF3, IL-1α, IL-10, IL-12 (p40), CXCL10, LIF, RANTES, CXCL9 and CCL4<br>• anti-ST2 treated group: concentrations of IL-5, IL-13, IL-6, CXCL1 and CCL4 were significantly reduced versus isotype group.<br>• BAL IL-9 was not significantly reduce	
				• Pulmonary resistance (RL)	• Increased baseline RL<br>> increased methacholine-induced changes in RL<br>> increased coefficients of lung tissue damping (G) and elastance (H)	

Author (year) Reference	Specie/ Analysis Unit	Exposure/ Dose/ route	Experimentation arm/groups	Outcomes	Findings	Conclusions
De Grove K.C, et al. 2016	Mice<br><br>Groups<br><br>*in vivo*<br>*ex vivo*	DEP, HDM<br>Dose:<br>DEP 25 mg<br>HDM 1 mg<br>DEP+HDM<br>Route:<br>Intranasal instillation days 1, 8, and 15	**C57BL/6 WT**<br>1,2,3,4 : DEP, HDM, DEP+HDM, Saline<br><br>**GATA3$^{+/nlslacz}$ (Gata-3-haploinsufficient)**<br>1,2,3,4 : DEP, HDM, DEP+HDM, Saline<br><br>**ROR$\alpha^{fl/fl}$ILR7^{Cre} (ILC2-deficient)**<br>1, 2: HDM, DEP+HDM<br><br>**ROR$\alpha^{fl/fl}$ILR7$^{+/+}$ (lack adaptive immune system)**<br>1, 2: HDM, DEP+HDM<br><br>**Rag2$^{-/-}$**<br>1,2,3,4 : DEP, HDM, DEP+HDM, Saline	• Airway inflammation	**DEP+HDM vs. other 3 groups in wild mice**<br>• Marked increase in epithelium-derived cytokines IL-25 and IL-33<br>• Increased numbers of DCs, neutrophils, ILC2s, CD41 T cells, CD81 T cells, and eosinophils.	Dysregulation of ILC2s and TH2 cells attenuates DEP-enhanced allergic airway inflammation. In addition, a crucial role for the adaptive immune system was shown on concomitant DEP+HDM exposure.
				• Type 2 cytokine production	• All ILC2s expressed ST2, resembling natural ILC2s<br>• BALF: increased IL-5 and IL-13 levels<br>• MLNs: markedly higher IL-4, IL-5,and IL-13 levels<br>• Serum: significantly increased HDM-specific IgG1 titers<br>• Presence of AHR	

Author (year) Reference	Specie/ Analysis Unit	Exposure/ Dose/ route	Experimentation arm/groups	Outcomes	Findings	Conclusions
Yang Qi, et al. 2016	Mice<br><br>Groups<br><br>*ex vivo*<br>*in vitro*	**O_3**<br>Dose:<br>3 ppm<br>Route:<br>Inhaled 2 hours on day 16<br>**Aspergillus fumigatus mixed with alum** for Allergen sensitization<br>Dose: 20 mg<br><br>Route:<br>Intraperitonea l injections on days 0 and 7<br>Intranasal on day 13	1. O_3 BALB/c<br>2. O3 C57BL/6<br>3. Air BALB/c<br>4. Air C57BL/6<br>5. O3 BALB/c treated with anti-Thy1.2 mAb<br>6. O3 BALB/c treated with anti-CD4 mAb	• BAL (Bronchoalveolar lavage) cell counts	O3 BALB/c vs other groups<br>• Enhanced A fumigatus–induced eosinophilia in BAL (doubled the numbers of eosinophils)	O3 exposure increased airway levels of IL-33, a potent activator of ILC2s.<br><br>Lung-resident ILC2s were the predominant early source of the Th2 cytokines IL-5 and IL-13 in O3-exposed mice.
				• ILC2s and RNA extraction from removed lungs	• Activates lung-resident ILC2<br>• Increased IL-5 by lung ILC2s in response to IL-33.<br>• Induced IL-33 mRNA activation and increased protein expression in the lung tissue in both strains.<br>• No ILC2 influx or proliferation within 12 hours after O3 exposure.<br>• ILC2s from the lungs: greater increased activation of Il5 and Il13 mRNA 12 hours after O3	
				• BAL IL-4 and IL-5 expression	• ILC2s O3 did not induce IL-5 or IL-13 production by CD41Thy11 TH cells isolated from the lungs 12 hours after exposure<br>• LC2s mediate O3-induced airway inflammation and AHR	
Kumagai K, et al. 2017	Mice<br><br>Groups<br><br>*in vivo*<br>*ex vivo*	O_3<br>Dose: 0.8 ppm<br>Route: Inhaled 1 day or 9 consecutive weekday (4 hr/day).	1. O3 C57BL/6 (ILC-sufficient Mice)<br>2. AirC57BL/6<br>3. O3 $Rag2^{-/-}$ (deficient Mice)<br>4. Air $Rag2^{-/-}$<br>5. O3 $Rag2^{-/-}Il2rg^{-/-}$ (depleted of all lymphoid cells including ILCs)<br>6. Air $Rag2^{-/-}Il2rg^{-/-}$	• Mucous Cell Metaplasia	O3 C57BL/6 vs other groups:<br>• Mucous cell metaplasia<br>• Greater volume densities of mucosubstances in airway epithelium lining this proximal large-diameter	Murine ILCs, but not T or B cells, play a crucial rule in ozone-induced mucous cell metaplasia, eosinophilic inflammation, and type 2 immunity in the lungs of mice.
				• Gene expression in type2 immune response	• Increased expression of genes involved in type 2 immune responses	
				• Number ILCs	• A small number of ILCs were present in ozone-exposed Rag2 /<br>Air- and ozone exposed Rag2 / mice had statistically similar numbers of lung ILC2s	

Author (year) Reference	Specie/ Analysis Unit	Exposure/ Dose/ route	Experimentation arm/groups	Outcomes	Findings	Conclusions
Burleson GR, et al. 1989	Fischer - 344 rats<br><br>Groups<br><br>*in vivo*<br>*in vitro*	O_3<br>**Dose:**<br>Continuous: 1,0 ppm 23,5 hours /day/ 1,3,7,o,10 consecutive days.<br>Doses response: 0,5 ppm or 0,1ppm<br>**Rout:** Ambient	1. O_3 day 1,<br>2. O_3 day 5<br>3. O_3 day 7<br>4. O_3 day 10.<br>5. Control: Clean air	NK cytotoxicity	Continuous exposure to 1.0 ppm ozone vs. clean air:<br>• 1, 5 or 7 days: immunosuppressive effect on pulmonary NK activity<br>• 10 days: The suppressed pulmonary NK activity returned to control levels	Ozone-induced suppression of pulmonary NK activity may be due to a direct effect on cells mediating NK activity. Ozone-suppressed pulmonary NK activity returned to normal levels in the continued presence of ozone inhalation. Suppression of NK activity by pollutant exposure may affect the ability of the host to defend against viral and neoplastic disease.
				Dose response studies	• NK activity was suppressed at 0.5 ppm ozone, but not at 0.1 ppm ozone, following 23.5 hours of exposure.	
				Total viable cell number, percent viability, and cell type	• No differences in percent viability of cells from whole-lung homogenate were observed for air- or ozone exposed groups at 1, 5, 7 or 10 days of exposure<br>• A decreased percentage of lymphocytes due to ozone exposure on day 5	
				Function of adherent cells	• Removal of adherent cells resulted in an enhanced NK activity in the non-adherent cell population for both air- and ozone-exposed animals compared to the percent lysis observed before removal of the adherent cells.<br>• Removal of adherent cells did not restore the ozone suppressed NK activity to control levels	
Lu X, et al. 2018	Mice<br><br>Groups<br><br>*in vivo*<br>*in vitro*	PM2.5<br>Dose:<br>suspension: 15 g/L PM2.5 intranasal	1. Control<br>2. OVA<br>3. OVA + PM2.5<br>4. OVA + PM2.5 + LXA4	ILC2-related transcription factors	OVA + $PM_{2.5}$ vs other groups<br><br>$PM_{2.5}$: Increased expression of RORα and GATA3 transcription factors.	PM2.5-induced inflammation plays a key role in the progression of asthma mice.

Author (year) Reference	Specie/ Analysis Unit	Exposure/ Dose/ route	Experimentation arm/groups	Outcomes	Findings	Conclusions
Finkelman F, et al. 2004	Mice<br>Groups<br>*in vivo*<br>*in vitro*	DEP<br>Dose:<br>10 mg/mL	1. DEP<br>2. Saline	Cytokine production	DEP vs. Saline<br>• DEP stimulate production of IL-6<br>• IFN-γ levels were considerably decreased, in some experiments decrease was 75% when mice were injected with DEP on 3 successive days	DEP potently inhibits IFN-γ production by NK and NKT cells, which is rapid in onset, long lasting, and dose related.<br>DEP induces an inhibitory effect on steady state INF-γ mRNA levels, and may also suppress INF-γ production through posttranscriptional mechanisms.
				TLRL4-induced IFN-γ production,	• Injection of 2 mg of DEP 2 h before LPS administration completely suppressed the LPS-induced IFN-γ response and partially suppressed LPS-induced IL-10 production, but had no effect on LPS induction of IL-6 or TNF production<br>• DEP suppressed the LPS-induced increase in IFN-γ mRNA (although not as completely as it suppressed IFN-γ secretion.	
				NK and NKT	• DEP suppressed IFN-γ production individual NK and NKT cells following LPS stimulation<br>• DEP failed to suppress anti-CD3 mAb-induced production of IFN- γ, or other cytokines, which are NKT cell derived.	
Zhao H, et al. 2014	Rats<br>Groups<br>*in vivo*<br>*in vitro*	$PM_{2.5}$<br>Dose:<br>1, 5, or 10mg/kg body weight<br>Route:<br>Intratracheal instillation<br><br>*S. aureus*	1. PM $_{2.5}$+ *S. aureus*<br>2. Control PBS+ *S. aureus*<br>3.	Number NK	$PM_{2.5}$ vs Control<br>• PM2.5:decreased NK cell response to subsequent S. aureus infection in the airway lumen<br>• PM2.5: decreased absolute NK cell number in BALF	• $PM_{2.5}$ increases susceptibility to respiratory infection by S. aureus.<br>• $PM_{2.5}$ decreases the number of NK cells in the lung and suppress AM phagocytosis which provide a potential mechanism to explain that associate ambient air pollution and pulmonary bacterial infections.
				Host defense restauration with adoptive transfer NK	• Prior PM2.5: increase in bacterial burden in the lung<br>• Adoptive NK cell transfer to the lung of previously PM2.5-exposed rats markedly reduced the bacterial burden	
				Potential mechanism by which NK cells contribute to host defense in pulmonary staphylococcal infection	• $PM_{2.5:}$ AMs co-cultured with NK cells demonstrated a higher rate of phagocytosis of S. aureus	

Author (year) Reference	Specie/ Analysis Unit	Exposure/ Dose/ route	Experimentation arm/groups	Outcomes	Findings	Conclusions
Müller L, et al. 2013a	Human *ex vivo* NK cells isolated from peripheral blood	DEP and pI:C Route: direct exposure of cell	1. DEP<br>2. pI:C<br>3. DEP+pI:C	Cytokine release	• pI:C significantly increased release of all cytokines (IL-1β, IL-2, IL-4, IL-8, IL-10, IL-12p70,IFN-γ, or TNF-α) tested other than IL-5 and IL-13<br>• DEP: little impact on cytokine release, other than a modest increase of IL-1β, IL8, and TNF-α.<br>• DEP+pI:C: induced increases were blunted for most cytokines, though this change was not statistically significant except for IL-4.	DEP reduced expression of the cytotoxic NK cell surface marker CD16, gene and protein expression of granzyme B and perforin, and the ability to kill target cells.
				Granzyme B and perforin expression	• pI:C: increased the expression of granzyme B and perforin compared to the vehicle control.<br>DEP: did not affect the RNA level of either granzyme B or perforin.<br>• pI: C+DEP-induced expression of granzyme B and granzyme B and perforin protein levels were significantly reduced	
				NK cell phenotype	• pI:C+DEP: expression of CD16, was reduced but the percentage of CD16- NK cells was increased<br>• DEP alone showed no effects on the NKp46 expression on NK cells	
Kucuksezer UC, et al. 2014	Human PBMC from peripheral blood cell *ex vivo*	O_3<br>Dose:<br>1, 5, 10, and 50 mg/mL cRPMI<br>Route: direct exposure of cell	1. O_3<br>2. unstimulated	NK number	O3 vs. unstimulated<br>• $O_3$1 mg/mL: increased total CD3- CD16+/56+ NK on both 3rd and 5th days of cell culture.<br>• No significant changes were observed among the expression levels of other surface molecules.	O_3 increased number of C16 cell and cytotoxicity.
				CD107a expression on NK cells Function	• O_3 1 and 5 mg/mL: induced increase in NK-cell cytotoxicity<br>• K562 cells induced a strong increase in the levels of CD107a expression<br>• Ozone exposure did not induce an increase of CD107a beyond K562-stimulated levels	

Author (year) Reference	Specie/ Analysis Unit	Exposure/ Dose/ route	Experimentation arm/groups	Outcomes	Findings	Conclusions
Müller L, et al. 2013b	Human NK from peripheral blood cell *ex vivo*	O_3<br>Dose:<br>0.4 ppm<br>Route:<br>Direct exposure of cell	1. O_3<br>2. Filtered air	Granzyme B expression	O_3 vs. Filtered air<br>• Intracellular granzyme B levels were lower in NK cells cocultured with NECs, albeit not statistically significant	O_3 reduced markers of activation, INF-γ production, and cytotoxic function.<br>O_3 upregulated ligands for NK in epithelial cells.
				Cytokine production	• O_3: decreased the expression of IFN- γ and enhanced, albeit not statistically significant, the expression of IL-4 in NK cells<br>• O_3-induced upregulation of ULBP3 and MICA/B on NECs mediates the suppression of IFN- γ.	
				Cell ligands to NK	suggest<br>O_3:alters the expression of NK cell ligands on NECs.	
				Cytotoxicity	O3: induced a reduction in NK cell cytotoxic function.	

References

Beamer CA, Girtsman TA, Seaver BP, Finsaas KJ, Migliaccio CT, Perry VK, et al. 2013. Il-33 mediates multi-walled carbon nanotube (mwcnt)-induced airway hyper-reactivity via the mobilization of innate helper cells in the lung. Nanotoxicology 7:1070-1081, DOI:10.3109/17435390.2012.702230.

Burleson GR, Keyes LL, JF S. 1989. Immunosuppression of pulmonary natural killer activity by exposure to ozone. Immunopharmacol Immunotoxicol 11:715-735, DOI:10.3109/08923978909005397.

De Grove KC, Provoost S, Hendriks RW, McKenzie ANJ, Seys LJM, Kumar S, et al. 2017. Dysregulation of type 2 innate lymphoid cells and Th2 cells impairs pollutant-induced allergic airway responses. J Allergy Clin Immunol 139:246-257 e244, DOI:10.1016/j.jaci.2016.03.044.

Finkelman FD, Yang M, Orekhova T, Clyne E, Bernstein J, Whitekus M, et al. 2004. Diesel exhaust particles suppress in vivo IFN- production by inhibiting cytokine effects on NK and NKT cells. J Immunol 172:3808-3813.

Kucuksezer UC, Zekiroglu E, Kasapoglu P, Adin-Cinar S, Aktas-Cetin E, Deniz G. 2014. A stimulatory role of ozone exposure on human natural killer cells. Immunol Invest 43:1-12, DOI:10.3109/08820139.2013.810240.

Kumagai K, Lewandowski RP, Jackson-Humbles DN, Buglak N, Li N, White K, et al. 2017. Innate lymphoid cells mediate pulmonary eosinophilic inflammation, airway mucous cell metaplasia, and type 2 immunity in mice exposed to ozone. Toxicol Pathol 45:692-704, DOI:10.1177/0192623317728135.

Lu X, Fu H, Han F, Fang Y, Xu J, Zhang L, et al. 2018. Lipoxin A4 regulates PM2.5-induced severe allergic asthma in mice via the Th1/Th2 balance of group 2 innate lymphoid cells. J Thorac Dis 10:1449-1459, DOI:10.21037/jtd.2018.03.02.

Mathews JA, Krishnamoorthy N, Kasahara DI, Cho Y, Wurmbrand AP, Ribeiro L, et al. 2017. IL-33 drives augmented responses to ozone in obese mice. Environ Health Perspect 125:246-253, DOI:10.1289/EHP272.

Müller L, Chehrazi CVE, Henderson MW, Noah TR, I J. 2013a. Diesel exhaust particles modify natural killer cell function and cytokine release. Part Fibre Toxicol 10:16, DOI:10.1186/1743-8977-10-16.

Müller L, Brighton LE, Jaspers I. 2013b. Ozone exposed epithelial cells modify cocultured natural killer cells. Am J Physiol Lung Cell Mol Physiol 304:L332-341, DOI:10.1152/ajplung.00256.2012.

Yang Q, Ge MQ, Kokalari B, Redai IG, Wang X, Kemeny DM, et al. 2016. Group 2 innate lymphoid cells mediate ozone-induced airway inflammation and hyperresponsiveness in mice. J Allergy Clin Immunology 137:571-578, DOI:10.1016/j.jaci.2015.06.037.

Zhao H, Li W, Gao Y, Li J, Wang H. 2014. Exposure to particular matter increases susceptibility to respiratory Staphylococcus aureus infection in rats via reducing pulmonary natural killer cells. Toxicol 325:180-188, DOI:10.1016/j.tox.2014.09.006.

Resource S3. Modified ARRIVE guidelines, and adapted scale from ARRIVE guidelines for experimental studies in human cells

Modified ARRIVE: Average score for 9 studies carried out in animals

			FULFILLMENT %
**TITLE**	1	Provide as accurate and concise a description of the content of the article as possible.	100
**ABSTRACT**	2	Provide an accurate summary of the background, research objectives, including details of the species or strain of animal used, key methods, principal findings and conclusions of the study.	94
**INTRODUCTION**			
**Background**	3	a. Include sufficient scientific background (including relevant references to previous work) to understand the motivation and context for the study, and explain the experimental approach and rationale.	100
		b. Explain how and why the animal species and model being used can address the scientific objectives and, where appropriate, the study's relevance to human biology.	50
**Objectives**	4	Clearly describe the primary and any secondary objectives of the study, or specific hypotheses being tested.	100
**METHODS**			
**Ethical statement**	5	Indicate the nature of the ethical review permissions, relevant licences (e.g. Animal [Scientific Procedures] Act 1986), and national or institutional guidelines for the care and use of animals, that cover the research.	88
**Study design**	6	For each experiment, give brief details of the study design including the number of experimental and control groups.	50
**Experimental procedures**	7	For each experiment and each experimental group, including controls, provide precise details of all procedures carried out. For example:	
		a. How (e.g. drug formulation and dose, site and route of administration, anaesthesia and analgesia used [including monitoring], surgical procedure, method of euthanasia). Provide details of any specialist equipment used, including supplier(s).	94
		b. When (e.g. time of day).	100
		c. Where (e.g. home cage, laboratory, water maze).	100
**In vitro procedures**	7a	a. Describe in detail all the in vitro procedures	100
		b. Describe in detail all the reagents, cells, kits including manufacturer used (manufacturer)	100
**Experimental animals**	8	a. Provide details of the animals used, including species, strain, sex, developmental stage (e.g. mean or median age plus age range) and weight (e.g. mean or median weight plus weight range).	88
		b. Provide further relevant information such as the source of animals, international strain nomenclature, genetic modification status (e.g. knock-out or transgenic), genotype, health/immune status, drug or test naïve, previous procedures, etc.	100
**Housing and husbandry**	9	Provide details of:	

		a. Housing (type of facility e.g. specific pathogen free [SPF]; type of cage or housing; bedding material; number of cage companions; tank shape and material etc. for fish).	77
		b. Husbandry conditions (e.g. breeding programme, light/dark cycle, temperature, quality of water etc for fish, type of food, access to food and water, environmental enrichment).	50
**Sample size**	10	a. Specify the total number of animals used in each experiment, and the number of animals in each experimental group.	50
		b. Explain how the number of animals was arrived at. Provide details of any sample size calculation used.	
**Allocating animals to experimental groups**	11	a. Give full details of how animals were allocated to experimental groups, including randomisation or matching if done.	50
**Experimental outcomes**	12	Clearly define the primary and secondary experimental outcomes assessed (e.g. cell death, molecular markers, behavioural changes).	100
**Statistical methods**	13	a. Provide details of the statistical methods used for each analysis.	72
		b. Specify the unit of analysis for each dataset (e.g. single animal, group of animals,<br>single neuron).	16
		c. Describe any methods used to assess whether the data met the assumptions of the statistical approach.	100
**RESULTS**			
**Baseline data**	14	For each experimental group, report relevant characteristics and health status of animals (e.g. weight, microbiological status, and drug or test naïve) prior to treatment or testing. (This information can often be tabulated).	0
**Numbers analysed**	15	a. Report the number of animals in each group included in each analysis. Report absolute numbers (e.g. 10/20, not 50%) (Schulz et al., 2010).	0
**Outcomes and estimation**	16	Report the results for each analysis carried out, with a measure of precision (e.g. standard error or confidence interval).	66,5
**DISCUSSION**			
**Interpretation/scientific implications**	18	a. Interpret the results, taking into account the study objectives and hypotheses, current theory and other relevant studies in the literature.	100
**Generalisability/translation**	19	Comment on whether, and how, the findings of this study are likely to translate to other species or systems, including any relevance to human biology.	70
**Funding**	20	List all funding sources (including grant number) and the role of the funder(s)<br>in the study.	80
**TOTAL**			74,9

Resource S4. Adapted scale for in *vitro* experiments in human cells (From ARRIVE guidelines).

Average score for 3 studies carried out in human cells

			FULFILLMENT %
**TITLE**	1	Provide as accurate and concise a description of the content of the article as possible.	100
**ABSTRACT**	2	Provide an accurate summary of the background, research objectives, including details of the species or strain of animal used, key methods, principal findings and conclusions of the study.	100
**INTRODUCTION**			
**Background**	3	a. Include sufficient scientific background (including relevant references to previous work) to understand the motivation and context for the study, and explain the experimental approach and rationale.	100
**Objectives**	4	Clearly describe the primary and any secondary objectives of the study, or specific hypotheses being tested.	100
**METHODS**			
**Ethical statement**	5	Indicate the nature of the ethical review permissions, written informed consent	100
**Study design**	6	For each experiment, give brief details of the study design including the number of experimental and control groups.	75
**In vitro procedures**	7	a. Describe in detail all the in vitro procedures	100
		b. Describe in detail all the reagents, cells, kits including manufacturer used (manufacturer)	100
**Sample size**	10	a. Specify the total number of subjects	100
		b. Explain how the number of animals was arrived at. Provide details of any sample size calculation used.	0
**Experimental outcomes**	12	Clearly define the primary and secondary experimental outcomes assessed (e.g. cell death, molecular markers, behavioural changes).	100
**Statistical methods**	13	a. Provide details of the statistical methods used for each analysis.	75
		b. Describe any methods used to assess whether the data met the assumptions of the statistical approach.	100
**RESULTS**			
**Baseline data**	14	For each experimental group, report relevant characteristics and health status of human subjects	0
**Outcomes and estimation**	16	Report the results for each analysis carried out, with a measure of precision (e.g. standard error or confidence interval).	100
**DISCUSSION**			
**Interpretation/scientific implications**	18	a. Interpret the results, taking into account the study objectives and hypotheses, current theory and other relevant studies in the literature.	100
**Funding**	20	List all funding sources (including grant number) and the role of the funder(s) in the study.	75
**TOTAL**			89.1

Resource 5S. List of the excluded studies

Christianson CA, Goplen NP, Zafar I, Irvin C, Good JT, Jr., Rollins DR, et al. Persistence of asthma requires multiple feedback circuits involving type 2 innate lymphoid cells and IL-33. J Allergy Clin Immunol. 2015;136(1):59-68 e14. DOI: 10.1016/j.jaci.2014.11.037.

Everaere L, Ait-Yahia S, Molendi-Coste O, Vorng H, Quemener S, LeVu P, et al. Innate lymphoid cells contribute to allergic airway disease exacerbation by obesity. J Allergy Clin Immunol. 2016;138(5):1309-18 e1. DOI:10.1016/j.jaci.2016.03.019.

Farhadi N, Lambert L, Triulzi C, Openshaw PJ, Guerra N. Culley FJ. Natural killer cell NKG2D and granzyme B are critical for allergic pulmonary inflammation. J Allergy Clin Immunol. 2014;133:827-35.DOI: 10.1016/j.jaci.2013.09.048.

Gold MJ, Antignano F, Halim TY, Hirota JA, Blanchet MR, Zaph C, et al. Group 2 innate lymphoid cells facilitate sensitization to local, but not systemic, TH2-inducing allergen exposures. J Allergy Clin Immunol. 2014;133(4):1142-8. DOI: 10.1016/j.jaci.2014.02.033.

Iijima K, Kobayashi T, Hara K, Kephart GM, Ziegler SF, McKenzie AN, et al. IL-33 and thymic stromal lymphopoietin mediate immune pathology in response to chronic airborne allergen exposure. J Immunol. 2014;193(4):1549-59. DOI:10.4049/jimmunol.1302984.

Kamijo S, Takeda H, Tokura T, Suzuki M, Inui K, Hara M, et al. IL-33-mediated innate response and adaptive immune cells contribute to maximum responses of protease allergen-induced allergic airway inflammation. J Immunol. 2013;190(9):4489-99. DOI:10.4049/jimmunol.1201212.

Klein Wolterink RG, Kleinjan A, van Nimwegen M, Bergen I, de Bruijn M, Levani Y, et al. Pulmonary innate lymphoid cells are major producers of IL-5 and IL-13 in murine models of allergic asthma. Eur J Immunol. 2012;42(5):1106-16. DOI:10.1002/eji.201142018.

Klein Jan A, Klein Wolterink RG, Levani Y, de Bruijn MJ, Hoogsteden HC, van Nimwegen M, et al. Enforced expression of GATA3 in T cells and group 2 innate lymphoid cells increases susceptibility to allergic airway inflammation in mice. J Immunol. 2014;192(4):1385-94. DOI:10.4049/jimmunol.1301888.

Li BW, de Bruijn MJ, Tindemans I, Lukkes M, KleinJan A, Hoogsteden HC, et al. T cells are necessary for ILC2 activation in house dust mite-induced allergic airway inflammation in mice. Eur J Immunol. 2016;46(6):1392-403. DOI:10.1002/eji.201546119.
